# Echocardiography in Pulmonary Arterial Hypertension: Comprehensive Evaluation and Technical Considerations

**DOI:** 10.3390/jcm10153229

**Published:** 2021-07-22

**Authors:** Weronika Topyła-Putowska, Michał Tomaszewski, Andrzej Wysokiński, Andrzej Tomaszewski

**Affiliations:** Department of Cardiology, Medical University of Lublin, 20-059 Lublin, Poland; weronika.topyla@gmail.com (W.T.-P.); tomaszewskimd@gmail.com (M.T.); andrzej.wysokinski@umlub.pl (A.W.)

**Keywords:** pulmonary arterial hypertension, echocardiography, three-dimensional, Doppler

## Abstract

Pulmonary arterial hypertension (PAH) is a rare, progressive disease in which there is a persistent, abnormal increase in pulmonary artery pressure. Symptoms of pulmonary hypertension are nonspecific and mainly associated with progressive right ventricular failure. The diagnosis of PAH is a multistep process and often requires the skillful use of several tests. The gold standard for the diagnosis of PAH is hemodynamic testing. Echocardiography currently plays an important role in the diagnostic algorithm of PAH as it is minimally invasive and readily available. Moreover, many echocardiographic parameters are closely related to pulmonary hemodynamics. It allows assessment of the right heart′s structure and function, estimation of the pressure in the right ventricle, right atrium, and pulmonary trunk, and exclusion of other causes of elevated pulmonary bed pressure. Echocardiographic techniques are constantly evolving, and recently, measurements made using new techniques, especially 3D visualization, have become increasingly important. In echocardiographic assessment, it is crucial to know current guidelines and new reports that organize the methodology and allow standardization of the examination. This review aims to discuss the different echocardiographic techniques used to evaluate patients with PAH.

## 1. Introduction

Pulmonary arterial hypertension (PAH) is a hemodynamic condition characterized by a persistent, abnormal increase in pulmonary artery pressure. Consequently, right ventricular heart failure develops, and symptoms such as dyspnea, fatigue, weakness, angina pain, and syncope appear [1]. The European Society of Cardiology has proposed the following clinical classification in which PAH is divided into the following subtypes: idiopathic, hereditary, associated with other diseases such as systemic connective tissue diseases or heart defects, as well as drug or toxin-induced [2,3].

PAH remains a chronic disease with an incompletely elucidated pathogenesis [4]. Clinical assessment of the patient plays a key role in selecting the treatment, observing the patient′s response to treatment, and potential escalation of the therapy [5,6]. Therapeutic decisions should be based on parameters that have a proven prognostic value [7]. Echocardiography plays an important role in the diagnosis of PAH as it is noninvasive and readily available. Moreover, many echocardiographic parameters are closely related to pulmonary hemodynamics [8]. Echocardiographic data provides a wealth of relevant information underpinning clinical management [9]. The purpose of this article is to describe the various echocardiographic techniques used to evaluate patients with PAH.

## 2. The Assessment of the Right Heart Cavities and Pericardial Dimensions Using Transthoracic Echocardiography

**Figure 1:**
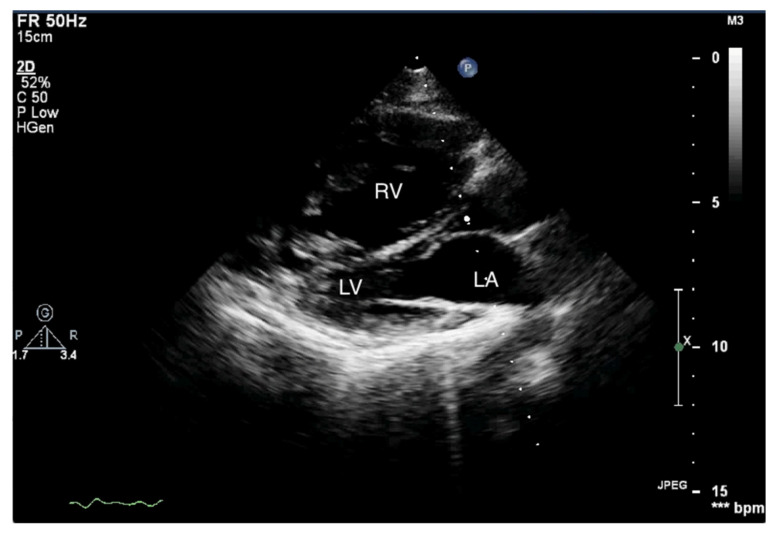
Enlarged right ventricle. Two-dimensional transthoracic echocardiography (2D-TTE), parasternal long-axis view (PLAX). RV: right ventricle, LV: left ventricle, LA: left atrium.

**Figure 2:**
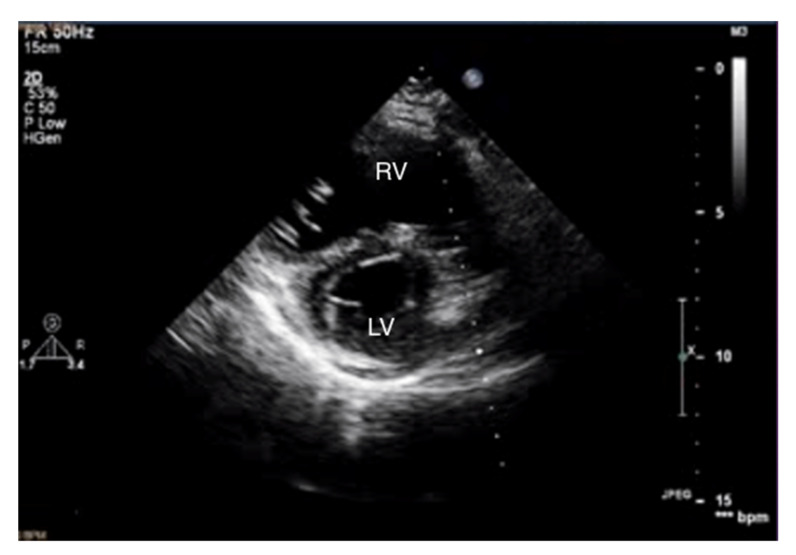
Enlarged right ventricle. 2D-TTE, parasternal short-axis view (PSAX). RV: right ventricle, LV: left ventricle.

When pulmonary circulation pressure increases in the course of PAH, the right ventricle (RV) is subjected to overload. Initially, it adapts to an increased vascular load by increasing muscle contraction force by up to 5-fold, thus maintaining normal stroke volume values. To maintain the increased contractility, the right ventricular muscle mass increases [10], and wall hypertrophy and RV cavity dilatation occur [11]. Thus, in the course of PAH, there are changes in the size ratio between the two ventricles. In advanced stages, the right ventricle is larger than the left (Figure 1 and Figure 2). Statistically, in patients with PAH, the RV is more dilated and functions worse than in other diseases characterized by pulmonary hypertension [12].

**Figure 3:**
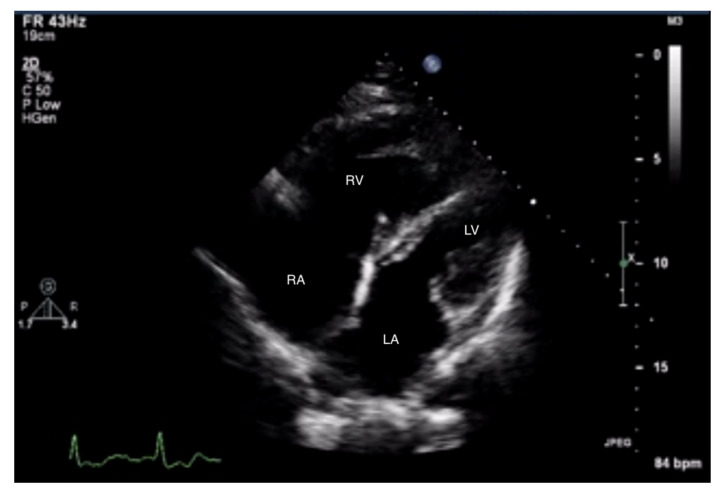
Enlarged right ventricle and right atrium. An abnormal size ratio between the right and left heart. 2D-TTE, 4CH view. 2D-TTE: two-dimensional transthoracic echocardiography; 4CH: four-chamber.

**Figure 4:**
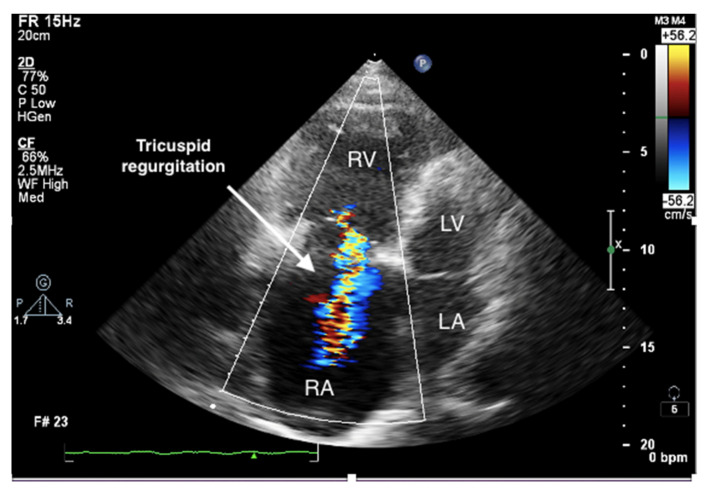
Enlarged right ventricle and right atrium. Tricuspid regurgitation. 2D-TTE, RV-focused apical 4CH view.

Measurements of both ventricles are recommended in the four-chamber (4CH) view, and the correct projection during measurements should be maintained (Figure 3 and Figure 4). In assessing the linear dimension of the right ventricle, the goal should be to obtain the maximum dimension of the right ventricle while preserving the visibility of the apex and the plane of section passing through the center of the LV. RV measurement in a five-chamber view with a visible left ventricular outflow tract is erroneous [13]. Under these measurement conditions, the normal RV size should not exceed 2/3 of the LV dimension [14].

Measurement of the RV-free wall is also important in the assessment of the long-term RV overload. This dimension should not exceed 0.5 cm in the end-diastolic phase [15]. Due to the irregular shape of the right ventricle, the assessment of its volume is difficult.

Given the complexity of the RV structure, three-dimensional echocardiography is extremely valuable. It allows for better visualization of the RV myocardial fibers and endocardial boundaries, and therefore a more accurate calculation of RV volume and ejection fraction [16]. However, obtaining an accurate 3D image of the right ventricle may be problematic. When using this technique, the manual selection of the relevant cardiac cycle phase is of particular importance, as well as the precise identification of endocardial margins and the inclusion of the tricuspid annulus.

Current studies suggest evaluating RV volume in relation to body surface area, which increases the diagnostic value of this parameter [17]. However, this technique requires extensive experience of the investigator and is not routinely used.

**Figure 5:**
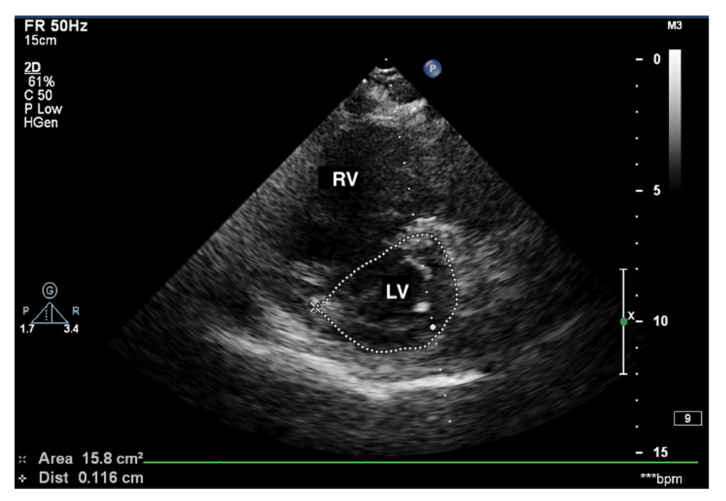
D-sign, enlarged right ventricle, ventricular septum displaced towards LV. 2D-TTE, short-axis view (SAX). LV: left ventricle; 2D-TTE: two-dimensional transthoracic echocardiography.

Transthoracic echocardiography (TTE) in a patient with advanced PAH is conspicuous by the RV cavity′s abnormal shape, especially in the parasternal short axis (Figure 5). The RV assumes a spherical shape, and the interventricular septum protrudes into the LV, forming the so-called D-shape sign [18]. If a D-shaped LV appears in the end-diastolic phase, it suggests RV pressure overload, whereas a D-sign shape in diastole suggests RV volume overload [19,20].

Another valuable parameter that determines the shape of the left ventricle is the left ventricular eccentricity index (EI), which is defined as the ratio of the anterior–inferior and septal–posterolateral cavity dimensions at the mid-ventricular level. A normal EI value does not exceed 1, and a measurement >1 indicates a flattened interventricular septum [21]. The results of this study showed that an EI ≥ 1.7, combined with a tricuspid annular plane systolic excursion (TAPSE) ≤ 15 mm, was associated with a higher rate of death and cardiac transplantation compared to patients with normal values [22].

**Figure 6:**
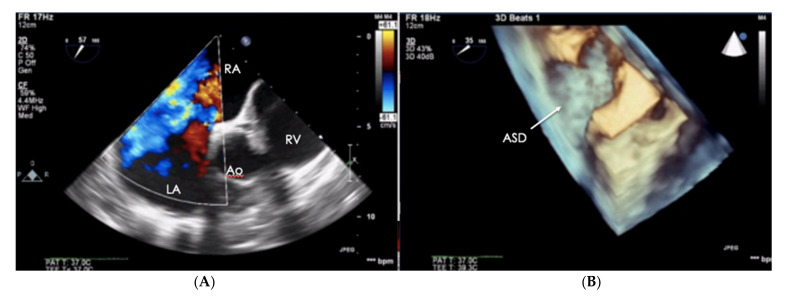
Patient with PAH-CHD–atrial septal defect. **(A)** 2D-TTE visualization, short-axis view (SAX). (**B**) The same defect visualized in the transthoracic echo (TTE), 3D visualization. PAH-CHD: pulmonary arterial hypertension-congenital heart disease; 2D-TTE: two-dimensional transthoracic echocardiography.

**Figure 7:**
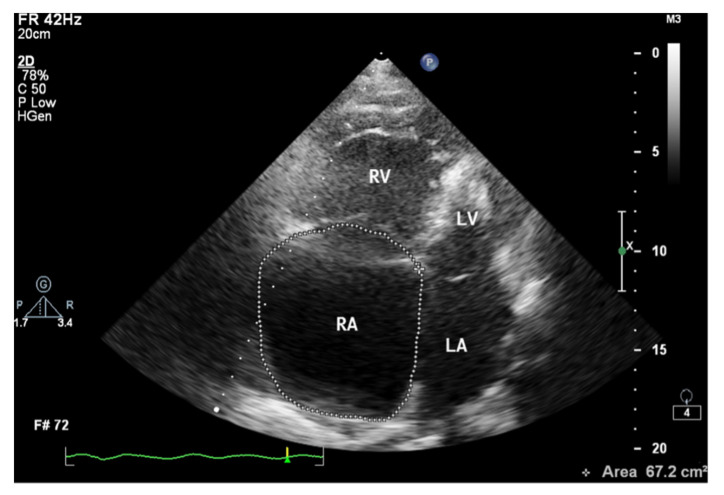
Enlarged right atrium with an area of 67.2 cm^2^. 2D-TTE, 4CH view. 2D-TTE: two-dimensional transthoracic echocardiography; 4CH: four-chamber.

When assessing the right heart′s size, the dimensions of the right atrium (RA) should not be overlooked (Figure 6). The linear dimension of RA should be marked in the 4CH view, perpendicular to the long axis of the heart (Figure 7) [23]. However, more valuable in terms of clinical significance is the RA volume, also measured in the 4CH view [23]—an RA area greater than 18 cm^2^ indicates enlargement and is one of the most common echo abnormalities found in PAH patients [24].

The assessment of the pericardial fluid is of utmost importance in echocardiography in patients with PAH. In a study of 81 patients with PAH, Raymond et al. demonstrated that pericardial effusion was a significant independent predictor of the adverse course of PAH and was associated with higher mortality [25]. Pericardial effusion can be assessed by 2D echocardiography, M mode, and Doppler analysis. However, four views (subcostal, four-chamber, and parasternal long and short axes) are recommended to determine the maximum thickness and precise location of the fluid [26]. Pericardial fluid in impending cardiac tamponade may be associated with the diastolic collapse of the right ventricle and left atrium, significant variability of inflow through the tricuspid valve (an increase of 50%), and mitral valve (a decrease of 25%) on the inhale; and decreased respiratory variation of the inferior vena cava due to increased central venous pressure [27]. However, in patients with advanced pulmonary hypertension, these features may be absent, even in cases where a large volume of pericardial fluid is present.

## 3. Right Ventricular Diastolic Dysfunction

As a result of RV overload, changes in the heart muscle occur at the macroscopic level and the level of myocytes. Increased tension, contractility, and hypertrophy of the RV muscle lead to an increased oxygen demand in its cells. The consequence of these changes is increased RV muscle stiffness, which is observed on echocardiography as an impaired RV diastolic function [28]. RV relaxation disorders are thought to precede contractile impairment in PAH patients and are important prognostic factors for PAH [29]. It has been shown that in patients with idiopathic PAH (IPAH), lateral mitral annular velocities correlate with pulmonary capillary wedge pressure (PCPW) while simultaneously excluding a left-heart-related cause of PAH [30].

In practice, the most commonly used parameters to assess RV diastolic function are the ratio of E- and A-waves of tricuspid inflow and the E-wave deceleration time. These values should be measured in the apical four-chamber view (4CH), preferably on expiration. Measurements during atrial fibrillation or in the presence of severe tricuspid regurgitation should be avoided, as the values obtained are unreliable [31].

Under normal conditions, early diastolic inflow predominates, i.e., E > A. When the RV is stiffened and less prone to stretch, changes in the E- and A-wave velocities of inflow across the tricuspid valve may occur [32]. Low E/A ratio values, especially <0.8, indicate impaired RV muscle relaxation [33]. As the RV muscle stiffens, end-diastolic pressure in the RV cavity increases, resulting in pseudonormalization of tricuspid inflow, i.e., a relative increase in early diastolic inflow. In contrast, E/A > 2 with a shortened E-wave deceleration time argues for even more advanced lesions [34].

An important technique in the evaluation of RV relaxation abnormalities is tissue Doppler imaging (TDI). It allows the determination of mitral and tricuspid annular velocities: diastolic (early diastolic E′ and after atrial contraction A′) and maximum systolic velocity (S′). Like the E and A waves of tricuspid inflow, under normal conditions, the early diastolic myocardial velocity wave predominates, and the lateral E′/A′ ratio is greater than 1 [35]. In impaired RV relaxation, this ratio is reversed [36]. However, in contrast to the tricuspid inflow wave velocities E and A, the E′/A′ ratio usually remains constant, i.e., <1 at different stages of the development of RV diastolic dysfunction [35].

## 4. Right Ventricular Systolic Dysfunction

**Figure 8:**
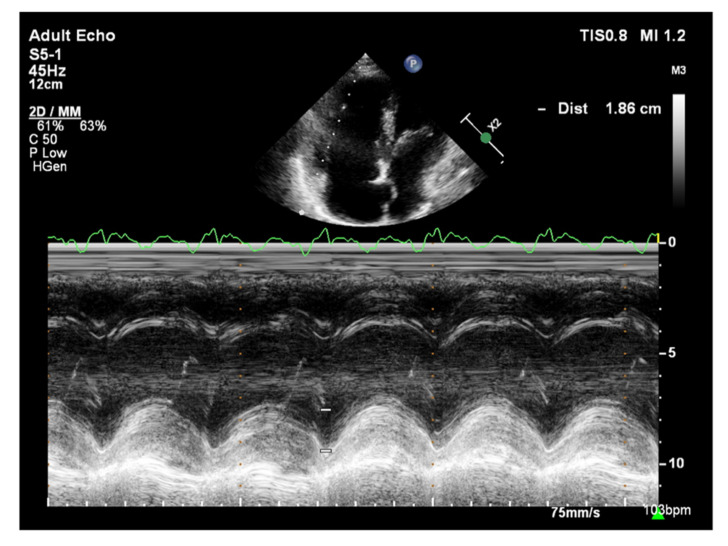
Patient with PAH, TAPSE = 1.86 cm. TTE, M-mode. PAH: pulmonary arterial hypertension; TAPSE: tricuspid annular plane systolic excursion; TTE: transthoracic echocardiography.

The longitudinal fibers of the right ventricular muscle are mainly responsible for muscle contraction. Contraction of these fibers also causes movement of the tricuspid valve annulus, which moves toward the ventricular apex in systole and toward the atrium in diastole [37]. Therefore, the amplitude of tricuspid annular systolic motion (TAPSE), obtained by the M-mode technique from the 4CH view, reflects RV systolic function (Figure 8) [37]. TAPSE values <18 mm are associated with poor prognosis and higher mortality in patients with PAH [38]. A correlation between TAPSE and RV ejection fraction (RVEF) measured by radionuclide angiography has also been demonstrated [39]. Considering that the TAPSE measurement is uncomplicated, highly reproducible, has little dependence on image quality, and has a high prognostic value, it is recommended that TAPSE is determined in all patients with PAH to assess RV systolic function [2]. However, TAPSE has a disadvantage of being angle-dependent and may be overestimated with apical rocking [40]. In addition, TAPSE may be load-dependent [41].

A more recent parameter for assessing RV systolic function is the aforementioned systolic tricuspid annular velocity (S′), evaluated using the TDI. This is obtained by placing the pulsed Doppler on the tricuspid valve annulus. Using TDI techniques, excellent projections can be obtained, however, the Doppler signal must be parallel to the direction of myocardial motion for reliable strain assessment [42]. A decrease in maximum tricuspid annular systolic velocity below 10 cm/s is closely associated with RV dysfunction, especially in young patients [43]. S′ values are, therefore, significantly reduced in PAH patients [44]. A correlation between a decrease in S′ velocity and a decrease in isotopically determining RVEF values in patients with chronic heart failure was also observed, where S′ < 11.5 cm/s corresponded to an RVEF < 45%.

Compared with LV, RV ejection fraction measurement by 2D technique is not applicable in everyday practice due to the complex spatial structure and extensive RV muscle fibers. Recent recommendations suggest the assessment of RV ejection fraction using a three-dimensional technique (RV 3D EF) [34]. For this purpose, it is recommended to use the automatic endocardial detection function. 3D ejection fraction measurements are comparable to those obtained using cardiac magnetic resonance (CMR) [45]. RV 3D EF values <45% are indicative of RV systolic dysfunction [46]. The limitations of this method are availability and dependence on the quality of imaging, difficulty in image acquisition with increased RV volumes, steady heart rate, and experience of the laboratory staff.

**Figure 9:**
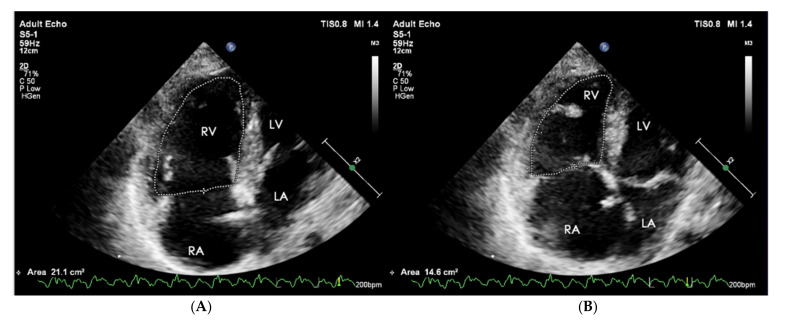
(**A**) End-diastolic volume RV = 21.1 cm^2^, (**B**) end-systolic volume RV = 14.6 cm^2^. FAC = 30.8%. FAC = A(diast) − A(sys)/A(dias) * 100%. 2D-TTE, 4CH view. RV: right ventricle; FAC: functional area change; 2D-TTE: two-dimensional transthoracic echocardiography; 4CH: four-chamber.

The equivalent of RVEF is the functional area change (FAC) of RV. It is a parameter of a proven clinical significance that has been recognized as a prognostic indicator of heart failure and sudden cardiac death in the course of PAH [47]. Its value is determined as a percentage after calculating the diastolic-systolic quotient of the difference in RV area and its diastolic area in the 4CH view (Figure 9). Normally it should exceed 35%, and lower values indicate RV systolic dysfunction [48].

The dP/dt parameter, an indicator of the rate of pressure rise during the systolic phase in the RV, has a documented value in assessing RV systolic function [49]. To measure the dP/dt parameter, it is necessary to visualize a clear contour of the tricuspid return wave using the continuous wave Doppler (CWD). Singbal et al. showed that the dP/dt ratio correlates strongly with RVEF measured by CMR. Values of dP/dt below 400 mmHg/s were consistent with reduced RVEF [50]. In addition, decreased dP/dt is a significant TAPSE-independent marker of an adverse course of PAH and CTEPH [51].

The Doppler-derived myocardial performance index (MPI/Tei Index) is a quantitative method used for the assessment of the global myocardial systolic and diastolic function in various disease entities, including the evaluation of the right ventricle function in patients with pulmonary arterial hypertension (PAH) [52]. It’s value is determined by the sum of RV isovolumetric contraction and diastolic time relative to the RV blood ejection time. Tei index has been shown to be significantly higher in patients with PAH, compared to controls [53]. Another study demonstrated that Tei index values correlate with PAP determined by cardiac hemodynamic testing in pediatric patients [54].

Its value does not depend on ventricular geometry, heart rate, preload, or the degree of tricuspid regurgitation [55]. The time points are determined from two Doppler flow recordings by the pulse wave method at the top of the tricuspid valve leaflets and RV ejection time (just below the pulmonary valve). When measured with PW, the index value should not exceed 0.43 [56]. In addition, MPI can be obtained using tissue Doppler imaging (TDI) which, records the motion of the tricuspid annulus. In this method, it is possible to make measurements in one projection and one cardiac cycle. MPI values using this method should be less than 0.54 [56]. This index should not be determined in patients with atrial fibrillation, aortic stenosis, and intraventricular conduction disorders [57,58]. Moreover, its disadvantages include the fact that the determination of the parameter requires high precision and is less reproducible.

## 5. Strain

**Figure 10:**
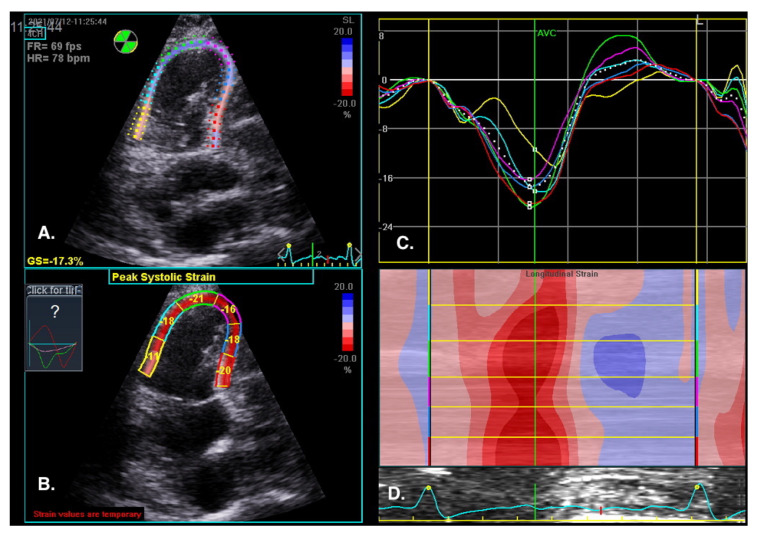
RV regional myocardial contractility (longitudinal strain) assessment by speckle tracking imaging. RV-focused view, 4CH. (**A**) Tracked apical loop with colour coding of the RV free wall and interventricular septum myocardial segments. (**B**) Regional end-systolic strain. (**C**) Segmental strain curves and segmental strain values displayed during the cardiac cycle. Global longitudinal strain at peak strain is visualized with the global strain curve (white dotted line). (**D**) M-mode representation of peak systolic strain. Blue colour- myocardial lengthening, red colour- myocardial shortening.

The visual assessment of myocardial contractility is very subjective and requires considerable experience from the investigator. New techniques can objectify the assessment of the segmental and global RV systolic function [59]. Myocardial strain is the percentage change in the distance between two points in the heart muscle during the cardiac cycle. In contrast, the strain rate is the rate of change in the distance between these points and is expressed in units of s^−1^. Initially, strain and strain rate were evaluated using TDI [60]. Currently, 2D strain and speckle tracking techniques are used, which involve automatic tracking of myocardial acoustic markers in standard echocardiographic images [61]. It allows the analysis of strain and strain rate in different directions (Figure 10). A newer and more precise technique for the assessment of strain and strain rate is three-dimensional echocardiography. This technology is currently under intensive development; however, reports of this method′s high efficacy in assessing the prognosis in patients with PAH are already available [62].

The normal RV systolic strain values in healthy subjects amount to: RV global strain −24.5 ± 3.8 and RV free wall strain −28.5 ± 4.8 [63].

The advantage of these methods is that they offer a very precise analysis of individual myocardial segments’ function, which enables visualization of discrete abnormalities, and detection of early stages of systolic dysfunction, often impossible to detect with conventional echocardiography [64].

Sachdev et al. demonstrated that RV longitudinal peak systolic strain (−15 ± 5%) and strain rate (−0.80 ± 0.29 s) are significantly reduced in patients with PAH. Moreover, RV-free wall strain was also reduced in the study group, which was associated with a decreased 1-year survival [65]. Other studies show that right atrial strain may also have a diagnostic value in patients with PAH [66].

A: Tracked apical loop with color coding of the RV-free wall and interventricular septum myocardial segments. B: Regional end-systolic strain. C: Segmental strain curves and segmental strain values displayed during the cardiac cycle. Global longitudinal strain at peak strain is visualized with the global strain curve (white dotted line). D: M-mode representation of peak systolic strain. The normal RV systolic strain values in healthy subjects amount to an RV global strain −24.5 ± 3.8 and RV free wall strain −28.5 ± 4.8 [63].

## 6. Echocardiographic Assessment of Right Heart Hemodynamic Parameters

The gold standard for assessing the hemodynamic parameters of the pulmonary circulation is to measure pressures in the right heart and pulmonary vessels during cardiac catheterization. While this test provides a diagnosis of PAH, it is invasive and more expensive. Therefore, among patients with suspected PAH, transthoracic echocardiography (TTE) is used as the first-choice test for noninvasive assessment of pulmonary vascular hemodynamics and initial estimation of RV, RA, and pulmonary trunk (PA) pressures [2,67].

**Figure 11:**
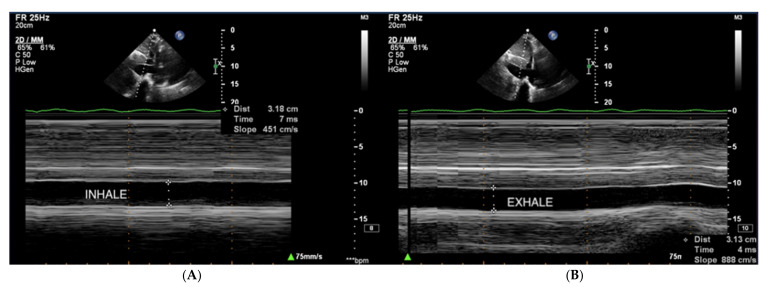
(**A**,**B**) IVC, no respiratory variability of IVC in the course of PAH. TTE, subcostal view. IVC: inferior vena cava; TTE: transthoracic echocardiography.

Right atrial pressure (RAP) is usually estimated based on the dimension and collapse rate of the inferior vena cava (IVC) in the subcostal view (Figure 11) [68]. The normal IVC width ranges between 15–21 mm, and IVC collapsibility on inspiration should exceed 50%. Moderately elevated pressure in RA exceeds 5 mmHg, and high pressure exceeds 10 mmHg [69].

**Figure 12:**
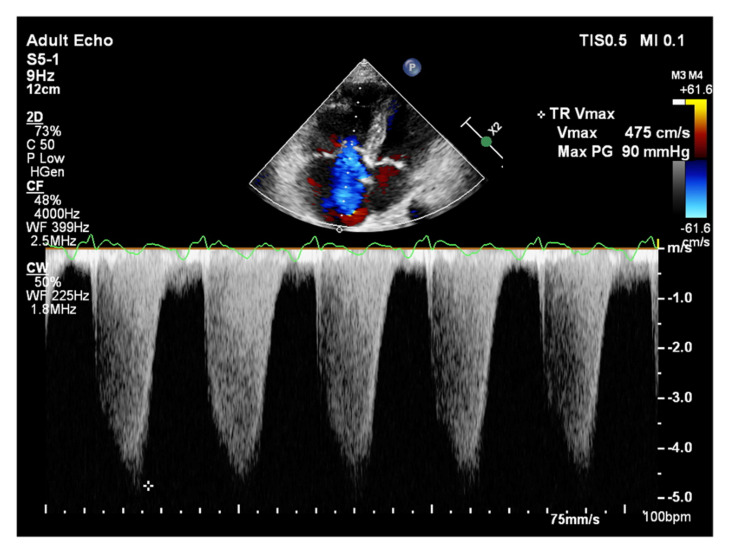
Doppler assessment of tricuspid regurgitant velocity (TRV). TTE, 4CH view. TTE: transthoracic echocardiography; 4CH: four-chamber.

**Figure 13:**
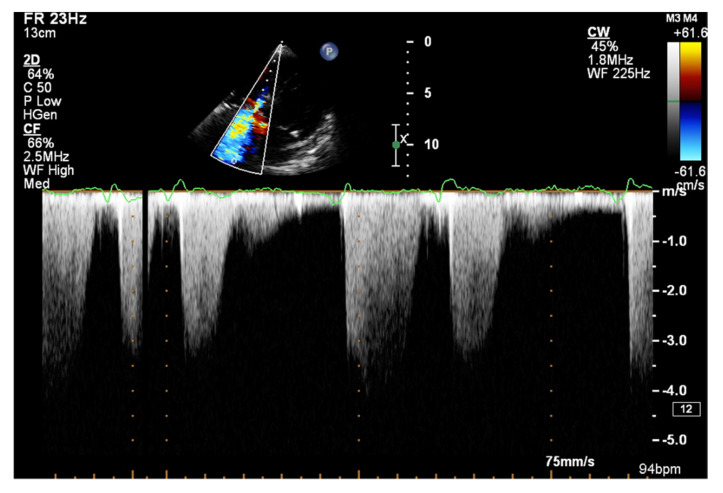
Doppler assessment of tricuspid regurgitant velocity (TRV). Differential rate of tricuspid regurgitation wave jets in atrial fibrillation. TTE, 4CH view. TTE: transthoracic echocardiography; 4CH: four-chamber.

It is believed that if there is no RV outflow tract stenosis, the right ventricular systolic pressure (RVSP) is equal to the pulmonary artery systolic pressure (PASP) [70]. In everyday practice, the calculation of PASP is based on a simplified Bernoulli equation applied to peak tricuspid regurgitation velocity (TRV). TRV should be measured in several views, aiming at the best image quality and maximum velocity in continuous-wave Doppler and avoiding excessive gain and artifacts (Figure 12 and Figure 13). According to the equation, PASP = 4 (TRV)2 + RAP [71,72]. In addition, based on the measurements of end-diastolic pulmonary regurgitant return wave velocity (PRVend), it is possible to estimate pulmonary artery diastolic pressure (PADP) using the PADP = 4 (PRVend)2 + RAP formula [73]. On the other hand, PASP and PADP values can be used to calculate approximations of mean pulmonary artery pressure (mPAP) using the mPAP = 1/3 (PASP) + 2/3 (PADP) formula [74]. Several other formulas for calculating mPAP can be found in the literature. Chemla et al. developed another method to calculate mPAP, according to the formula mPAP = 0.61 × PASP + 2 mmHg [75].

Another independent method of estimating mPAP is based on the measurement of acceleration time (AcT) of pulmonary flow by pulsed-wave Doppler, measured in the RV outflow tract. This is the time necessary for blood flowing from the RV to the pulmonary artery to reach maximum velocity. The higher the pulmonary artery pressure, the faster this velocity is reached and, therefore, the shorter the AcT [76]. Data suggests that AcT may be more sensitive than TRV in detecting early or latent pulmonary vascular impedance changes [77]. However, for mPAP estimates to be reliable, AcT should exceed 100 msec. Furthermore, due to other variables, including obesity, cardiac index, and left-right atrial leakage, this parameter is not widely used to calculate mPAP. In contrast, it plays an important role in assessing pulmonary vascular bed resistance, especially in the diagnosis of acute pulmonary embolism [78].

## 7. Conclusions

Echocardiography examination provides important prognostic data in the assessment of pulmonary vascular hemodynamics and right heart load. The most important parameters help to estimate the mean pressure in the right ventricle, right atrium, and pulmonary artery. The examination should also include the assessment of RV systolic and diastolic function. Everyday clinical practice indicates that a simple and reproducible TAPSE measurement is most commonly performed in this group of patients. The use of new measurement techniques such as TDI and 3D visualization is recommended for this purpose. The combined consideration of several echocardiographic parameters describing RV systolic and diastolic function increases their prognostic value. The assessment of the size of the heart chambers and linear values should include parameters that take into account the RA and RV area. In the case of parameters dependent on height and weight or gender, indexing is recommended.

## Data Availability

Not applicable.

## References

[1] Maron B.A., Brittain E.L., Hess E., Waldo S.W., Barón A.E., Huang S., Goldstein R.H., Assad T., Wertheim B.M., Alba G.A. (2020). Pulmonary vascular resistance and clinical outcomes in patients with pulmonary hypertension: A retrospective cohort study. Lancet Respir. Med..

[2] Galie N., Humbert M., Vachiery J.L., Gibbs S., Lang I., Torbicki A., Simonneau G., Peacock A., Vonk Noordegraaf A., Beghetti M. (2016). 2015 ESC/ERS guidelines for the diagnosis and treatment of pulmonary hypertension: The joint task force for the diagnosis and treatment of pulmonary hypertension of the european society of cardiology (ESC) and the european respiratory society (ERS): Endorsed by: Association for european paediatric and congenital cardiology (AEPC), international society for heart and lung transplantation (ISHLT). Eur. Heart J..

[3] Simonneau G., Montani D., Celermajer D.S., Denton C.P., Gatzoulis M.A., Krowka M., Williams P.G., Souza R. (2019). Haemodynamic definitions and updated clinical classification of pulmonary hypertension. Eur. Respir. J..

[4] Hasan B., Tuyghun E., Yang Y., Tuerxun P., Li X. (2020). Comprehensive network analysis to identify the molecular pathogenesis of pulmonary hypertension. Minerva Cardioangiol..

[5] Yaghi S., Novikov A., Trandafirescu T. (2020). Clinical update on pulmonary hypertension. J. Investig. Med..

[6] Sachpekidis V., Karvounis H., Giannakoulas G. (2018). The role of echocardiography in the diagnostic work-up of pulmonary hypertension. Cont. Cardiol. Educ..

[7] Miotti C., Papa S., Manzi G., Scoccia G., Luongo F., Toto F., Malerba C., Cedrone N., Sciomer S., Ciciarello F. (2021). The Growing Role of Echocardiography in Pulmonary Arterial Hypertension Risk Stratification: The Missing Piece. J. Clin. Med..

[8] Ferrara F., Zhou X., Gargani L., Wierzbowska-Drabik K., Vriz O., Fadel B.M., Stanziola A.A., Kasprzak J., Vannan M., Bossone E. (2019). Echocardiography in Pulmonary Arterial Hypertension. Curr. Cardiol. Rep..

[9] Ni J.R., Yan P.J., Liu S.D., Hu Y., Yang K.H., Song B., Lei J.Q. (2019). Diagnostic accuracy of transthoracic echocardiography for pulmonary hypertension: A systematic review and meta-analysis. BMJ Open.

[10] Spruijt O.A., de Man F.S., Groepenhoff H., Oosterveer F., Westerhof N., Vonk-Noordegraaf A., Bogaard H.J. (2015). The effects of exercise on right ventricular contractility and right ventricular-arterial coupling in pulmonary hypertension. Am. J. Respir. Crit. Care Med..

[11] Vonk Noordegraaf A., Westerhof B.E., Westerhof N. (2017). The Relationship Between the Right Ventricle and its Load in Pulmonary Hypertension. J. Am. Coll. Cardiol..

[12] Grapsa J., Gibbs J.S., Dawson D., Watson G., Patni R., Athanasiou T., Punjabi P.P., Howard L.S.G.E., Nihoyannopoulos P. (2012). Morphologic and functional remodeling of the right ventricle in pulmonary hypertension by real time three dimensional echocardiography. Am. J. Cardiol..

[13] Galderisi M., Cosyns B., Edvardsen T., Cardim N., Delgado V., Di Salvo G., Donal E., Sade L.E., Ernande L., Garbi M. (2017). 2016–2018 EACVI Scientific Documents Committee. Standardization of adult transthoracic echocardiography reporting in agreement with recent chamber quantification, diastolic function, and heart valve disease recommendations: An expert consensus document of the European Association of Cardiovascular Imaging. Eur. Heart J. Cardiovasc. Imaging.

[14] Kim J., Srinivasan A., Seoane T., Di Franco A., Peskin C.S., McQueen D.M., Paul T.K., Feher A., Geevarghese A., Rozenstrauch M. (2016). Echocardiographic Linear Dimensions for Assessment of Right Ventricular Chamber Volume as Demonstrated by Cardiac Magnetic Resonance. J. Am. Soc. Echocardiogr..

[15] Dutta T., Aronow W.S. (2017). Echocardiographic evaluation of the right ventricle: Clinical implications. Clin. Cardiol..

[16] Lakatos B.K., Tokodi M., Kispál E., Merkely B., Kovács A. (2020). Morphological and Functional Assessment of the Right Ventricle Using 3D Echocardiography. J. Vis. Exp..

[17] Eslami M., Larti F., Larry M., Molaee P., Badkoobeh R.S., Tavoosi A., Safari S., Parsa A.F. (2017). Two-dimensional echocardiographic right ventricle measurements adjusted to body mass index and surface area in a normal population. J. Clin. Ultrasound.

[18] Ferrando-Castagnetto F., Ricca-Mallada R., Selios V., Ferrando R. (2017). Atrial Arrhythmias and Scintigraphic “D-shape” Sign in Pulmonary Artery Hypertension. World J. Nucl. Med..

[19] Weyman A.E., Wann S., Feigenbaum H., Dillon J.C. (1976). Mechanism of abnormal septal motion in patients with right ventricular volume overload. A cross sectional echocardiographic study. Circulation.

[20] Farmakis I.T., Demerouti E., Karyofyllis P., Karatasakis G., Stratinaki M., Tsiapras D., Athanassopoulos G., Voudris V., Giannakoulas G. (2021). Echocardiography in Pulmonary Arterial Hypertension: Is It Time to Reconsider Its Prognostic Utility?. J. Clin. Med..

[21] Howard L.S., Grapsa J., Dawson D., Bellamy M., Chambers J.B., Masani N.D., Nihoyannopoulos P., Simon R., Gibbs J. (2012). Echocardiographic assessment of pulmonary hypertension: Standard operating procedure. Eur. Respir. Rev..

[22] Ghio S., Klersy C., Magrini G., D’Armini A.M., Scelsi L., Raineri C., Pasotti M., Serio A., Campana C., Viganò M. (2010). Prognostic relevance of the echocardiographic assessment of right ventricular function in patients with idiopathic pulmonary arterial hypertension. Int. J. Cardiol..

[23] Whitlock M., Garg A., Gelow J., Jacobson T., Broberg C. (2010). Comparison of left and right atrial volume by echocardiography versus cardiac magnetic resonance imaging using the area-length method. Am. J. Cardiol..

[24] Austin C., Alassas K., Burger C., Safford R., Pagan R., Duello K., Kumar P., Zeiger T., Shapiro B. (2015). Echocardiographic assessment of estimated right atrial pressure and size predicts mortality in pulmonary arterial hypertension. Chest.

[25] Raymond R.J., Hinderliter A.L., Willis P.W., Ralph D., Caldwell E.J., Williams W., Ettinger N.A., Hill N.S., Summer W.R., de Boisblanc B. (2002). Echocardiographic predictors of adverse outcomes in primary pulmonary hypertension. J. Am. Coll. Cardiol..

[26] Pérez-Casare A., Cesar S., Brunet-Garcia L., Sanchez-de-Toledo J. (2017). Echocardiographic Evaluation of Pericardial Effusion and Cardiac Tamponade. Front Pediatr..

[27] Alerhand S., Carter J.M. (2019). What echocardiographic findings suggest a pericardial effusion is causing tamponade?. Am. J. Emerg. Med..

[28] Rain S., Handoko M.L., Trip P., Gan C.T., Westerhof N., Stienen G.J., Paulus W.J., Ottenheijm C.A., Marcus J.T., Dorfmüller P. (2013). Right ventricular diastolic impairment in patients with pulmonary arterial hypertension. Circulation.

[29] Forfia P.R., Vachiéry J.L. (2012). Echocardiography in pulmonary arterial hypertension. Am. J. Cardiol..

[30] Ruan Q., Nagueh S.F. (2007). Clinical application of tissue Doppler imaging in patients with idiopathic pulmonary hypertension. Chest.

[31] Huttin O., Voilliot D., Mandry D., Venner C., Juillière Y., Selton-Suty C. (2016). All you need to know about the tricuspid valve: Tricuspid valve imaging and tricuspid regurgitation analysis. Arch. Cardiovasc. Dis..

[32] Bernardo R.J., Haddad F., Couture E.J., Hansmann G., de Jesus Perez V.A., Denault A.Y., de Man F.S., Amsallem M. (2020). Mechanics of right ventricular dysfunction in pulmonary arterial hypertension and heart failure with preserved ejection fraction. Cardiovasc. Diagn. Ther..

[33] DiLorenzo M.P., Bhatt S.M., Mercer-Rosa L. (2015). How best to assess right ventricular function by echocardiography. Cardiol. Young.

[34] Zaidi A., Knight D.S., Augustine D.X., Harkness A., Oxborough D., Pearce K., Ring L., Robinson S., Stout M., Willis J. (2020). Education Committee of the British Society of Echocardiography. Echocardiographic assessment of the right heart in adults: A practical guideline from the British Society of Echocardiography. Echo. Res. Pract..

[35] Pietrzak R., Werner B. (2014). Right ventricular function assessment using tissue Doppler imaging and speckle tracking echocardiography. J. Ultrason..

[36] Caballero L., Kou S., Dulgheru R., Gonjilashvili N., Athanassopoulos G.D., Barone D., Baroni M., Cardim N., Gomez de Diego J.J., Oliva M.J. (2015). Echocardiographic reference ranges for normal cardiac Doppler data: Results from the NORRE Study. Eur. Heart J. Cardiovasc. Imaging.

[37] Hammarstrom E., Wranne B., Pinto F.J., Puryear J., Popp R.L. (1991). Tricuspid annular motion. J. Am. Soc. Echocardiogr..

[38] Forfia P.R., Fisher M.R., Mathai S.C., Housten-Harris T., Hemnes A.R., Borlaug B.A., Chamera E., Corretti M.C., Champion H.C., Abraham T.P. (2006). Tricuspid annular displacement predicts survival in pulmonary hypertension. Am. J. Respir. Crit. Care Med..

[39] Aepfelbacher F.C., Yeon S.B., Ho K.K., Parker J.A., Danias P.G. (2003). ECG-gated 99mTc single-photon emission CT for assessment of right ventricular structure and function: Is the information provided similar to echocardiography?. Chest.

[40] Motoji Y., Tanaka H., Fukuda Y., Sano H., Ryo K., Sawa T., Miyoshi T., Imanishi J., Mochizuki Y., Tatsumi K. (2016). Association of apical longitudinal rotation with right ventricular performance in patients with pulmonary hypertension: Insights into overestimation of tricuspid annular plane systolic excursion. Echocardiography.

[41] Li Y., Wang Y., Yang Y., Liu M., Meng X., Shi Y., Zhu W., Lu X. (2018). Tricuspid annular displacement measured by 2-dimensional speckle tracking echocardiography for predicting right ventricular function in pulmonary hypertension: A new approach to evaluating right ventricle dysfunction. Medicine.

[42] Ng A.C., Thomas L., Leung D.Y. (2010). Tissue Doppler echocardiography. Minerva Cardioangiol..

[43] Kukulski T., Hubbert L., Arnold M., Wranne B., Hatle L., Sutherland G.R. (2000). Normal regional right ventricular function and its change with age: A Doppler myocardial imaging study. J. Am. Soc. Echocardiogr..

[44] You X.D., Pu Z.X., Peng X.J., Zheng S.Z. (2007). Tissue Doppler imaging study of right ventricular myocardial systolic activation in subjects with pulmonary arterial hypertension. Chin. Med. J..

[45] Sugeng L., Mor-Avi V., Weinert L., Niel J., Ebner C., Steringer-Mascherbauer R., Bartolles R., Baumann R., Schummers G., Lang R.M. (2010). Multimodality comparison of quantitative volumetric analysis of the right ventricle. JACC Cardiovasc. Imaging.

[46] Maffessanti F., Muraru D., Esposito R., Gripari P., Ermacora D., Santoro C., Tamborini G., Galderisi M., Pepi M., Badano L.P. (2013). Age-, body size-, and sex-specific reference values for right ventricular volumes and ejection fraction by three-dimensional echocardiography: A multicenter echocardiographic study in 507 healthy volunteers. Circ. Cardiovasc. Imaging.

[47] Anavekar N.S., Skali H., Bourgoun M., Ghali J.K., Kober L., Maggioni A.P., McMurray J.J., Velazquez E., Califf R., Pfeffer M.A. (2008). Usefulness of right ventricular fractional area change to predict death, heart failure, and stroke following myocardial infarction (from the VALIANT ECHO Study). Am. J. Cardiol..

[48] Rudski L.G., Lai W.W., Afilalo J., Hua L., Handschumacher M.D., Chandrasekaran K., Solomon S.D., Louie E.K., Schiller N.B. (2010). Guidelines for the echocardiographic assessment of the right heart in adults: A report from the American Society of Echocardiography endorsed by the European Association of Echocardiography, a registered branch of the European Society of Cardiology, and the Canadian Society of Echocardiography. J. Am. Soc. Echocardiogr..

[49] Anconina J., Danchin N., Selton-Suty C., Isaaz K., Juilliere Y., Buffet P., Edel F., Cherrier F. (1993). Noninvasive estimation of right ventricular dP/dt in patients with tricuspid valve regurgitation. Am. J. Cardiol..

[50] Singbal Y., Vollbon W., Huynh L.T., Wang W.Y., Ng A.C., Wahi S. (2015). Exploring Noninvasive Tricuspid dP/dt as a Marker of Right Ventricular Function. Echocardiography.

[51] Ameloot K., Palmers P.J., Vande Bruaene A., Gerits A., Budts W., Voigt J.U., Delcroix M. (2014). Clinical value of echocardiographic Doppler-derived right ventricular dp/dt in patients with pulmonary arterial hypertension. Eur. Heart J. Cardiovasc. Imaging.

[52] Yücel M., Alp H., Yorulmaz A., Karaarslan S., Baysal T. (2019). Prediction of the development of pulmonary arterial hypertension with Tei Index in congenital heart diseases with left-to-right shunt. Turk. Kardiyol. Dern Ars..

[53] Tei C., Dujardin K.S., Hodge D.O., Bailey K.R., McGoon M.D., Tajik A.J., Seward S.B. (1996). Doppler echocardiographic index for assessment of global right ventricular function. J. Am. Soc. Echocardiogr..

[54] Dyer K.L., Pauliks L.B., Das B., Shandas R., Ivy D., Shaffer E.M., Valdes-Cruz L.M. (2006). Use of myocardial performance index in pediatric patients with idiopathic pulmonary arterial hypertension. J. Am. Soc. Echocardiogr..

[55] Cahill J.M., Horan M., Quigley P., Maurer B.J., McDonald K. (2002). Doppler echocardiographic indices of diastolic function in heart failure admissions with preserved left ventricular systolic function. Eur J. Heart Failure.

[56] Ogihara Y., Yamada N., Dohi K., Matsuda A., Tsuji A., Ota S., Ishikura K., Nakamura M., Ito M. (2014). Utility of right ventricular Tei-index for assessing disease severity and determining response to treatment in patients with pulmonary arterial hypertension. J. Cardiol..

[57] Sud S., Massel D. (2009). An echocardiographic study of the limitations of the Tei index in aortic stenosis. Echocardiography.

[58] Lakoumentas J.A., Panou F.K., Kotseroglou V.K., Aggeli K.I., Harbis P.K. (2005). The Tei index of myocardial performance: Applications in cardiology. Hellenic, J. Cardiol..

[59] Lee J.H., Park J.H. (2018). Strain Analysis of the Right Ventricle Using Two-dimensional Echocardiography. J. Cardiovasc. Imaging.

[60] Heimdal A., Støylen A., Torp H., Skjaerpe T. (1998). Real-time strain rate imaging of the left ventricle by ultrasound. J. Am. Soc. Echocardiogr..

[61] Ayach B., Fine N.M., Rudski L.G. (2018). Right ventricular strain: Measurement and clinical application. Curr. Opin. Cardiol..

[62] Li Y., Wang T., Haines P., Li M., Wu W., Liu M., Chen Y., Jin Q., Xie Y., Wang J. (2020). Prognostic Value of Right Ventricular Two-Dimensional and Three-Dimensional Speckle-Tracking Strain in Pulmonary Arterial Hypertension: Superiority of Longitudinal Strain over Circumferential and Radial Strain. J. Am. Soc. Echocardiogr..

[63] Morris D.A., Krisper M., Nakatani S., Köhncke C., Otsuji Y., Belyavskiy E., Radha Krishnan A.K., Kropf M., Osmanoglou E., Boldt L.H. (2017). Normal range and usefulness of right ventricular systolic strain to detect subtle right ventricular systolic abnormalities in patients with heart failure: A multicentre study. Eur. Heart J. Cardiovasc. Imaging.

[64] Shukla M., Park J.H., Thomas J.D., Delgado V., Bax J.J., Kane G.C., Howlett J.G., White J.A., Fine N.M. (2018). Prognostic Value of Right Ventricular Strain Using Speckle-Tracking Echocardiography in Pulmonary Hypertension: A Systematic Review and Meta-analysis. Can. J. Cardiol..

[65] Sachdev A., Villarraga H.R., Frantz R.P., McGoon M.D., Hsiao J.F., Maalouf J.F., Ammash N.M., McCully R.B., Miller F.A., Pellikka P.A. (2011). Right ventricular strain for prediction of survival in patients with pulmonary arterial hypertension. Chest.

[66] Bhave N.M., Visovatti S.H., Kulick B., Kolias T.J., McLaughlin V.V. (2017). Right atrial strain is predictive of clinical outcomes and invasive hemodynamic data in group 1 pulmonary arterial hypertension. Int. J. Cardiovasc. Imaging.

[67] Thenappan T., Ormiston M.L., Ryan J.J., Archer S.L. (2018). Pulmonary arterial hypertension: Pathogenesis and clinical management. BMJ.

[68] Beigel R., Cercek B., Luo H., Siegel R.J. (2013). Noninvasive evaluation of right atrial pressure. J. Am. Soc. Echocardiogr..

[69] Rein A.J., Lewis N., Forst L., Gotsman M.S., Lewis B.S. (1982). Echocardiography of the inferior vena cava in healthy subjects and in patients with cardiac disease. Isr. J. Med. Sci..

[70] Yock P.G., Popp R.L. (1984). Noninvasive estimation of right ventricular systolic pressure by Doppler ultrasound in patients with tricuspid regurgitation. Circulation.

[71] Giardini A. (2011). Limitations inherent to the simplified Bernoulli equation explain the inaccuracy of Doppler echocardiographic estimates of pulmonary artery pressures in patients with pulmonary hypertension. Chest.

[72] Amsallem M., Sternbach J.M., Adigopula S., Kobayashi Y., Vu T.A., Zamanian R., Liang D., Dhillon G., Schnittger I., McConnell M.V. (2016). Addressing the Controversy of Estimating Pulmonary Arterial Pressure by Echocardiography. J. Am. Soc. Echocardiogr..

[73] Masuyama T., Kodama K., Kitabatake A. (1986). Continuous-wave Doppler echocardiographic detection of pulmonary regurgitation and its application to noninvasive estimation of pulmonary artery pressure. Circulation.

[74] Aduen J.F., Castello R., Lozano M.M., Hepler G.N., Keller C.A., Alvarez F., Safford R.E., Crook J.E., Heckman M.G., Burger C.D. (2009). An alternative echocardiographic method to estimate mean pulmonary artery pressure: Diagnostic and clinical implications. J. Am. Soc. Echocardiogr..

[75] Hemla D., Castelain V., Provencher S., Humbert M., Simonneau G., Hervé P. (2009). Evaluation of various empirical formulas for estimating mean pulmonary artery pressure by using systolic pulmonary artery pressure in adults. Chest.

[76] Yared K., Noseworthy P., Weyman A.E., McCabe E., Picard M.H., Baggish A.L. (2011). Pulmonary artery acceleration time provides an accurate estimate of systolic pulmonary arterial pressure during transthoracic echocardiography. J. Am. Soc. Echocardiogr..

[77] Pavelescu A., Vanderpool R., Vachiéry J.L., Grunig E., Naeije R. (2012). Echocardiography of pulmonary vascular function in asymptomatic carriers of BMPR2 mutations. Eur. Respir. J..

[78] Kurzyna M., Torbicki A., Pruszczyk P., Burakowska B., Fijałkowska A., Kober J., Oniszh K., Kuca P., Tomkowski W., Burakowski J. (2002). Disturbed right ventricular ejection pattern as a new Doppler echocardiographic sign of acute pulmonary embolism. Am. J. Cardiol..

